# The FAST-AIMS Clinical Mass Spectrometry Analysis System

**DOI:** 10.1155/2009/598241

**Published:** 2009-07-09

**Authors:** Nafeh Fananapazir, Alexander Statnikov, Constantin F. Aliferis

**Affiliations:** ^1^Department of Biomedical Informatics, Vanderbilt University, Nashville, TN 37232, USA; ^2^Cincinnati Children's Hospital Medical Center, Cincinnati, OH 45229, USA; ^3^Center for Health Informatics and Bioinformatics, New York University, NY 10016, USA

## Abstract

Within clinical proteomics, mass spectrometry analysis of biological samples is emerging as an important high-throughput technology, capable of producing powerful diagnostic and prognostic models and identifying important disease biomarkers. As interest in this area grows, and the number of such proteomics datasets continues to increase, the need has developed for efficient, comprehensive, reproducible methods of mass spectrometry data analysis by both experts and nonexperts. We have designed and implemented a stand-alone software system, FAST-AIMS, which seeks to meet this need through automation of data preprocessing, feature selection, classification model generation, and performance estimation. FAST-AIMS is an efficient and user-friendly stand-alone software for predictive analysis of mass spectrometry data. The present resource review paper will describe the features and use of the FAST-AIMS system. The system is freely available for download for noncommercial use.

## 1. Background

Mass spectrometry is a widely used analytical technique capable of discriminating proteins and their postdigestion peptide products on the basis of mass and charge. Within the last several years, researchers have explored the use of mass spectrometry for clinical applications in the broad areas of early cancer detection, clinical diagnosis, and clinical outcome prediction. Published reports indicate remarkable potential for this technology to diagnose disease and predict its outcome with minimally invasive testing procedures, low cost, and—in some cases—with unprecedented accuracy. It is expected that mass spectrometry together with other high-throughput technologies will lead the way to personalized medicine in the near future [1–4].

The analysis of high-throughput data such as gene expression microarray has involved a number of statistical challenges, the most notable of which include large number of predictor variables and small sample sizes of typical datasets, high information redundancy, limited reproducibility, and multiple sources of noise in the data [5–7]. The analysis of mass spectrometry data introduces further analytic challenges, requiring additional preprocessing steps such as baseline correction of spectra, detection of peaks corresponding to proteins and their peptide products, normalization of the intensity values of these peaks, alignment or calibration of peaks across spectra, and superimposition of intensity values corresponding to the same mass-to-charge values [4, 7–11]. Furthermore, in typical mass spectrometry analysis, proteins are unknown a priori and identified by mass-to-charge ratios (as opposed to the use of known features in microarray analysis); hence, data modelling tends to be heavily, if not exclusively, guided by the data and not by prior biological knowledge [12].

Contrary to microarray data analysis that is nowadays facilitated by a multitude of software systems for assisting both experts and inexperienced analysts [13], no such system currently exists for mass spectrometry data that will *automatically * enable a statistically naïve user to (i) create, from start to finish, diagnostic/outcome prediction models, (ii) estimate their predictive performance in future applications, and (iii) select protein markers. Out of existing systems for analysis of mass spectrometry data, a commercial platform ProTS Marker by Biodesix (http://www.biodesix.com) allows to *manually* perform the tasks mentioned above, however, it requires significant expertise of the user in analysis of proteomics data. Therefore, there is a strong need for systems that will allow both high-quality first-pass analyses of mass spectrometry data as well as enhancing work of the data analyst.

In the present report, we describe a software system FAST-AIMS (Fully Automated Software Tool for Artificial Intelligence in Mass Spectrometry) which aims at providing this functionality. In addition, given the current lack of protocol standardization for such proteomic data analysis, FAST-AIMS offers an intuitive, stepwise sequence of analysis in aid of developing such standardization. The system is a stand-alone application that runs on a Windows computer and can work with mass spectrometry data generated by different instruments, for example, MALDI, SELDI, LC-MS/MS, and so forth.

## 2. System Description

The FAST-AIMS system consists of a command-line executable (implemented in Matlab and C) and a wizard-like graphic user interface (implemented in Borland Delphi) that calls the above executable with different parameters. Novice users should utilize a wizard-like interface, while more experienced analysts may benefit from command-line interface for the batch analyses. FAST-AIMS runs on a Windows computer and requires neither Matlab nor other software to be installed. The need for Maltab is circumvented by using Matlab Compiler that translated all relevant Matlab code into C.

**Figure 1:**
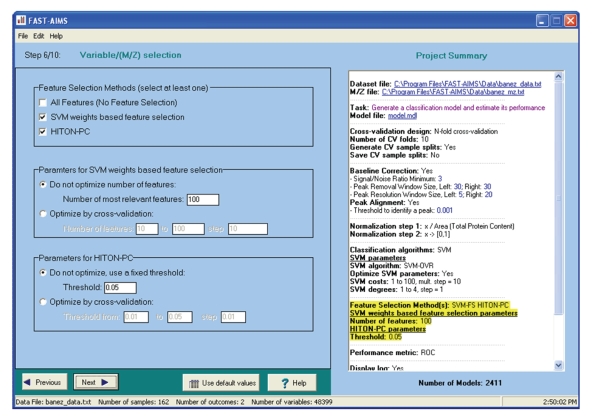
*Screenshot of the FAST-AIMS system*. The left part of the screen contains options for the current analysis step (variable (M/Z) selection). The summary of the entire analysis project is shown in the right part of the screen.

FAST-AIMS possesses an intuitive wizard-like interface (Figure 1) with defaults provided so that users need not be familiar with all steps of data analysis. Given a mass spectrometry dataset as input, FAST-AIMS can automatically perform one of the following tasks: (a) generate a classification model by optimizing the parameters of analysis algorithms to ensure its optimal performance; (b) obtain an unbiased estimate of the future classification performance of the optimized model; (c) generate a model and estimate classification performance in tandem; (d) apply an existing model to a new set of samples. In the process, the system also offers the option of identifying biomarkers/features that capture the classification tasks of interest and can be used to explore the underlying biological mechanisms. All FAST-AIMS functions follow the nested N-fold cross-validation design that allows optimization of predictive model parameters while providing an unbiased classification performance estimate [13, 14]. Below, we outline the main steps in the analysis as performed by FAST-AIMS.

1. An opening window asks whether the user wishes to start a new analysis or load a previously saved analysis. In starting a new analysis, the user first selects the location of a mass spectrometry data file. The system recognizes space delimited text data files, each row of which corresponds to the intensity values of a sample and the first column of which corresponds to the response/outcome of each sample. The user can optionally specify the location of the text file with M/Z values arranged in a column. The latter file is used for preprocessing of mass spectrometry data and for generation of the analysis report.
2. The user is then asked to choose from one of the four analysis tasks outlined above.
3. Next, the user selects cross-validation design: N-fold cross-validation or leave-one-out cross-validation.
4. Then the user is asked to select whether baseline subtraction and peak detection [15] and/or peak alignment [16] should be performed.
5. The user specifies a normalization sequence to be applied to the data.
6. The classifier and its parameters are selected by the user. The system uses support-vector machine (SVM) classification algorithms [17] because of their high performance in published analyses of mass spectrometry data and other types of high-throughput data, most notably gene expression arrays in which SVMs outperformed all major pattern recognition algorithms [18, 19].
7. Feature/variable selection algorithms (and their parameters) are selected by the user. The algorithms include: using all features in the data, HITON-PC [20], and SVM weight-based feature selection [21].
8. The user selects a performance estimation metric: either area under ROC curve [22], accuracy, or an entropy-based metric relative classifier information (RCI) [23].
9. A log file destination is specified by the user.
10. A report file destination is specified by the user.
11. The user clicks “Run”. If the task involves generation of classification model and/or performance estimation, the system considers each permutation of the classifier and feature selection algorithm parameters.

Some steps can be performed on each spectrum independently (e.g., peak detection, baseline subtraction, and some normalization methods). All steps that require consideration of multiple spectra (e.g., peak alignment, classification, and feature selection) are performed on each training set of the data to ensure unbiased estimates of the classification performance.

## 3. Discussion

In our prior work [24], we have evaluated an early prototype version of the FAST-AIMS system using an SELDI mass spectrometry prostate cancer dataset with 162 spectra. The evaluation revealed that the system achieves classification performance comparable to the human expert statistician and superior to the previously published analysis in literature. It took users on average 30 minutes to familiarize with functionality of the system and 19 hours to build the final model, while an expert statistician spent 7 hours for this task. Application of the model to new samples took less than 5 minutes. In general, depending on the dataset characteristics and selected data analysis options, it can take FAST-AIMS from 5 minutes to several days to analyze a dataset (i.e., build the final model). However, a basic first-pass analysis can be conducted within 2–3 hours for a dataset with 200 samples and 60.000 features. Compared to the early prototype version of FAST-AIMS used in [24], the current system has additional functionality (e.g., more performance estimation metrics, ability to output report in HTML file, automatically updated project summary and estimation of complexity of the analysis, built-in help system) and stable software code ready for the public release. Furthermore, the present report provides FAST-AIMS to the community for the first time.

The FAST-AIMS system is designed for mass spectrometry data analysis and inherits challenges of using that technology. For example, given a mass spectrometry dataset a system identifies biomarkers/features that correspond to anonymous peak intensities and may have little significance for the clinical practice [25, 26]. Any potentially interesting peak would have to be examined using an MS/MS system and the findings have to be confirmed by a quantitative proteomics approach [27, 28]. However, we emphasize that FAST-AIMS can be universally applied to various MS and tandem MS measurement technologies and does not require use of specific proteomic platforms such as SELDI or MALDI.

Finally, we believe that recent efforts of the cancer Biomedical Informatics Grid (caBIG) would allow improving the future versions of the FAST-AIMS system. For example, FAST-AIMS can incorporate proteomics related data standards such as mzData (for capturing peak list information) and mzXML (for storage and exchange of mass spectroscopy data) which would make the system more interoperable and would allow obtaining data directly from the proteomics instruments.

## 4. Conclusions

In this report, we have presented a software system FAST-AIMS for automated predictive analysis of mass spectrometry data. FAST-AIMS is a stand-alone Windows application with an intuitive wizard-like interface that guides the user through different steps of data analysis. The system is freely available for download for noncommercial use from http://www.dsl-lab.org/FAST-AIMS.

**Authors' Contributions**

All authors conceived and designed the software. NF and AS implemented the software. NF and AS drafted the paper. All authors read, revised, and approved the final manuscript.
